# Structural Basis for Human PECAM-1-Mediated Trans-homophilic Cell Adhesion

**DOI:** 10.1038/srep38655

**Published:** 2016-12-13

**Authors:** Menglong Hu, Hongmin Zhang, Qun Liu, Quan Hao

**Affiliations:** 1 School of Biomedical Sciences, University of Hong Kong, Laboratory Block, 21 Sassoon Road, Pokfulam, Hong Kong, China; 2 Department of Biology and Shenzhen Key Laboratory of Cell Microenvironment, Southern University of Science and Technology, Shenzhen 518055, China; 3 Biology Department, Brookhaven National Laboratory, Upton, NY 11973, USA

## Abstract

Cell adhesion involved in signal transduction, tissue integrity and pathogen infection is mainly mediated by cell adhesion molecules (CAM). One CAM member, platelet–endothelial-cell adhesion molecule-1 (PECAM-1), plays an important role in tight junction among endothelia cells, leukocyte trafficking, and immune response through its homophilic and heterophilic binding patterns. Both kinds of interactions, which lead to endogenous and exogenous signal transmission, are derived from extracellular immunoglobulin-like (IgL) domains and cytoplasmic immunoreceptor tyrosine-based inhibitory motifs (ITIMs) of PECAM-1. To date, the mechanism of trans-homophilic interaction of PECAM-1 remains unclear. Here, we present the crystal structure of PECAM-1 IgL1-2 trans-homo dimer. Both IgL 1 and 2 adopt the classical Ig domain conformation comprised of two layers of β-sheets possessing antiparallel β-strands with each being anchored by a pair of cysteines forming a disulfide bond. The dimer interface includes hydrophobic and hydrophilic interactions. The Small-Angle X-ray Scattering (SAXS) envelope of PECAM-1 IgL1-6 supported such a dimer formation in solution. Cell adhesion assays on wildtype and mutant PECAM-1 further characterized the structural determinants in cell junction and communication.

Platelet–endothelial-cell adhesion molecule-1 (PECAM-1), also well known as CD31, is a 130 kilodalton protein which belongs to both type I transmembrane glycoprotein and immunoglobulin (Ig) superfamily^1^. PECAM-1 can be divided into three parts: extracellular region which contains six immunoglobulin-like (IgL) domains with nine potential N-glycosylation sites, transmembrane region with one single α helix, and cytoplasmic region which possesses two separate tyrosine residues (Y663 and Y686) described as immunoreceptor tyrosine-based inhibitory motifs (ITIMs)^2^,^3^. Its distribution primarily concentrates on endothelial cells and platelets^1^,^3^. Furthermore, PECAM-1 is also expressed by those cells responsible for innate immunity, such as monocytes, neutrophils, and natural killer cells, and for adaptive immunity B and T cells^4^,^5^,^6^,^7^,^8^. In addition, it can be expressed either by dendritic cells acting as antigen presenting cells or by certain vascular tumor cells^3^,^9^.

As a cell adhesion molecule, PECAM-1 functions principally in cell junction but also plays a role in leukocyte trafficking and immune response^2^,^10^,^11^. Among adjacent endothelial cells, PECAM-1 interacts with homogeneous molecules in a trans-homophilic binding manner that does not rely on transmembrane and cytoplasmic regions. This kind of homophilic interaction can be interrupted efficaciously by IgL1 and IgL1–2 antibodies both *in vivo* and *in vitro*^12^,^13^. The homo-binding pattern leads to phosphorylation on Y663 and Y686 which triggers tertiary complex formation consisting of PECAM-1/SHP-2/β-catenin^14^. Phosphorylated ITIMs recruit SHP-2 which will further dephosphorylate β-catenin. Subsequently, β-catenin re-associates with another adhesion molecule VE-cadherin to stabilize cell junction^15^,^16^,^17^. In addition to its positive feedback, the PECAM-1 homophilic binding intensifies cell junction and gives rise to a tight barrier of endothelium. In the course of diapedesis inclusive of homo- and hetero- interactions of PECAM-1, its crosstalk with integrin α_v_β_3_ establishes adhesion between leukocyte and endothelia cells as well as association between cell adhesion molecules and integrins including VCAM-1/α_4_ and ICAM/β_2_^18^,^19^,^20^. Moreover, PECAM-1 could associate with CD38 on lymphocytes and CD177 on neutrophils and may work in the regulation of diapedesis according to previous studies^21^,^22^. In contrast, PECAM-1 homophilic interactions can stimulate formation of lateral border recycling compartment (LBRC) in endothelial cells to promote transmigration of leukocytes^23^. Meanwhile, between naïve T-cells and antigen presenting cells, PECAM-1 homophilic interaction mediated phosphorylation on ITIMs trigger ERK1/2 downstream signal pathways via SHP-2 recruitment followed by gene transcription for cell survival, resulting in enhanced tolerance of naïve T-cells^9^. Furthermore, PECAM-1 can interplay with *Plasmodium falciparum* erythrocyte membrane protein 1 (PfEMP-1) expressed by *Plasmodium falciparum* infected red blood cells, which could potentially accelerate infected cell aggregation in blood vessels^24^,^25^.

Although numerous important physiological functions of PECAM-1 root in homophilic and heterophilic interactions on its extracellular six IgL domains, little molecular mechanism is known except the recent reported IgL1–2 structure by Zhu and co-workers^26^. However, this structure was a cis-dimer in one asymmetric unit with the IgL2 “fold-out” as opposed to classical immunoglobulin. Here, we report a crystal structure of trans-homophilic IgL1-2 dimer to reveal the binding pattern of the IgL1-2 homo-interactions and to provide mechanistic understanding of IgL1-2 in cell adhesion.

## Results

### Structure determination

**Figure 1:**
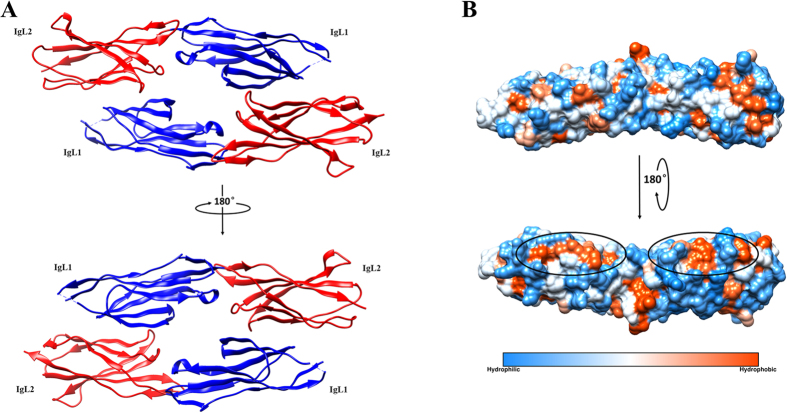
Overall structure of PECAM-1 IgL1-2 trans-homophilic dimer. (**A**) Front and back views of the IgL1-2 trans-homophilic dimer structure in which IgL1 and IgL2 are labeled with blue and red colors respectively. IgL1 interacts with IgL2 from the opposite monomer. Both IgL1 and IgL2 hold a traditional immunoglobulin conformation that consists of two β-sheet layers anchored by one disulfide bond. (**B**) Hydrophobic and hydrophilic surface of an IgL1-2 monomer. Hydrophobicity surfaces are presented by *Chimera* with orange red for most hydrophobic and dodger blue for most hydrophilic^33^. The two interface regions of the IgL1-2 dimerization are highlighted by two black ellipses. Hydrophobic areas are concentrated at the center of each region and hydrophilic areas form two circumambient barriers that surround hydrophobic centers.

The extracellular portions of PECAM-1 on adjacent cell surfaces show a homophilic binding pattern, which contributes to tight cell junction and builds a barrier between basal lamina and blood vessel together with other cell adhesion molecules^12^,^13^. Previous studies have demonstrated that the first two IgL domains of PECAM-1 were involved in the homophilic interaction. Therefore, we chose the fragment spanning residues 28-232 (IgL1-2) for crystallographic study. To avoid the uncontrolled glycosylation in insect cell expression system that might impair crystallization, we mutated three potential glyco-sites on IgL1-2 (N52Q, N84Q and N151Q). Then we expressed, crystallized this mutant IgL1-2 and obtained the native diffraction data set. However, we could not solve the phase problem by using existing immunoglobulin structures as search models despite high similarity among immunoglobulin superfamily members. Thus, IgL1-2 was split into individual IgL1 and IgL2 spanning residues 28-132 and 133-232 respectively. These two small fragments were expressed in *Escherichia coli*. We crystallized both native and Se-Met IgL1 fragments and collected diffraction data sets of high resolution at 2.0 Å. The experimental phases of the SeMet IgL1 data set were determined by single-wavelength anomalous dispersion (SAD) method^27^. Based on the partial structural model of IgL1, we used molecular replacement and then several cycles of manual model rebuilding and refinement to phase the native IgL1-2 data set. The whole structure of IgL1-2 was finally determined to 3.0 Å resolution where one asymmetric unit contains two IgL1-2 molecules (chains A and B) forming a dimer (Fig. 1A).

### Overall structure

The homodimer of PECAM-1 IgL1-2 displays a trans-binding pattern that relies on the interactions between IgL1 and IgL2 derived from two opposite molecules. Both IgL1 and IgL2 are highly similar to each other and their conformations belong to the classical architecture of immunoglobulin. Each of them is composed of two parallel β sheet layers anchored by a pair of cysteine residues (Cys57, 109 and Cys152, 206 on IgL1 and 2 respectively) which form a disulfide bond whereby every β sheet layer contains 3-5 antiparallel β strands. Moreover, a short α helix (residues 100-103) of unknown function links the 7^th^ and 8^th^ β strands of IgL1.

### Homophilic adhesion interface

**Figure 2:**
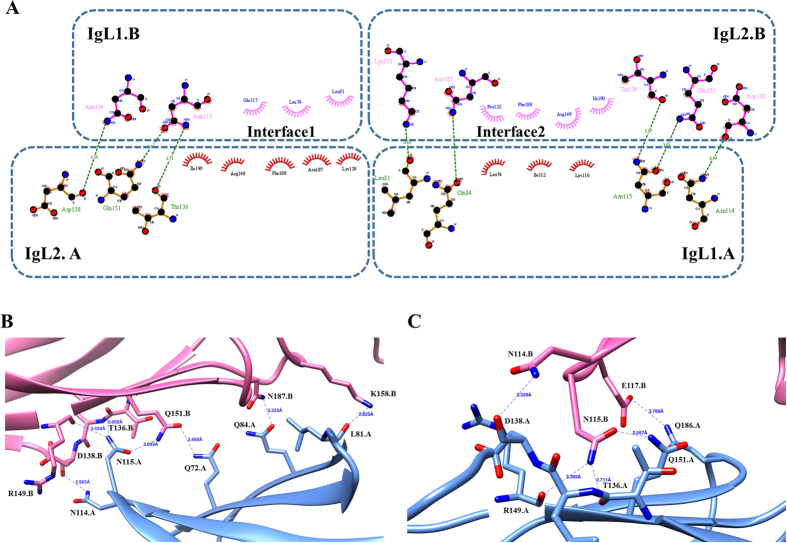
Interfaces of IgL1-2 dimerization. (**A**) Residues that participate in homophilic interaction are plotted and displayed in 2D with *DIMPLOT* of the LigPlot plus suite^35^. Protein side chains primarily involved in hydrophilic interaction are shown as ball-and-stick. Red, blue, black balls represent oxygen, nitrogen and carbon atoms respectively. Green dotted lines labeled with distances indicate hydrogen bonds. Non-bonded residues contributing to hydrophobic interactions are shown as spoked arcs. Oxford-blue dotted lines embrace residues located at the same IgL domains. Interface2 (in between IgL2-B and IgL1-A) and Interface1 (in between IgL1-B and IgL2-A) correspond to (**B**) and (**C**) revealing 3D representation of hydrogen bonds processed by *Chimera*. Chain A and B are colored in corn flower blue and hot pink respectively. O (red) and N (blue) atoms that contribute to hydrogen bonds are linked by dotted lines (dark blue) with distance labels.

As shown in Fig. 1, the two IgL1-2 fragments are packed to each other in a face-to-face antiparallel pattern with the side face of one β sheet. There are two interacting surfaces, one between IgL1 of chain A (IgL1-A) and IgL2 of chain B (IgL2-B) and the other between IgL2 of chain A (IgL2-A) and IgL1 of chain B (IgL1-B). After analyzing the interaction surface of IgL1-2 dimer (Fig. 1B), the solvent accessible areas buried in interfaces on chain A and B are 1079.0 Å^2^ and 1094.2 Å^2^ respectively, and there are two types of reciprocities consisting of hydrophobic interaction and hydrogen bond. Although the overall conformation of the two interfaces is almost identical, there are subtle differences between them. On one hand, there are two hydrophobic interaction surfaces and each of them is located between the vis-à-vis IgL1 and IgL2 from opposite molecules (Fig. 2A). The two hydrophobic centers are mainly composed of nonpolar residues with participation of several C and O atoms on surrounding polar residues. On interface1 (IgL2-A-IgL1-B), residues including K158, N187, F188 from IgL2-A and L74, L81, E117 from IgL1-B form the core of the hydrophobic interactions, while N115 from IgL1-B forms satellite hydrophobic interactions with I190, R149 from IgL2-A by donating C and O atoms on backbone to reinforce the hydrophobic contact. Similarly, interface2 (IgL1A-IgL2-B) residues including L74, I112, K116 from IgL1-A and R149, P155, F188, I190 from IgL2-B form the hydrophobic center while the C/O atoms from N115, Q84 on IgL2-A and N187 on IgL1-B form satellite hydrophobic interactions. Among those residues, L74, I112, F188 and I190 were proven to affect the intermolecular binding pattern of PECAM-1 *in vivo* by our mutational analysis described below. On the other hand, twelve pairs of residues form hydrogen bonds with donor-acceptor distances of 2.56-3.77 Å (Fig. 2B and C). On IgL1-A, Q72, L81, Q84, N114 and N115 form hydrogen bonds with Q151, K158, N187, D138 and T136/R149/Q151 on IgL2-B respectively (Fig. 2B). Meanwhile, N114, N115 and E117 on IgL1-B interplay with D138, T136/R149/Q151 and Q186 on IgL2-A respectively (Fig. 2C). Since we mutated N52, N84, and N151 residues to Q to eliminate glycosylation and the side chain of N is shorter than Q, there are eight hydrogen bonds expected with donor-acceptor distances of 2.71–3.77 Å. after excluding four resulted from mutated residues. Thus, the hydrophobic binding core surrounded by hydrogen bonds gives rise to the homophilic dimerization of IgL1-2.

### SAXS verification of PECAM-1 trans-homophilic binding pattern

To eliminate crystal packing artifact of IgL1-2, wild type full length extracellular domain of PECAM-1 (IgL1-6), which contains nine potential glycosylation sites, was expressed in the same insect cell system that produced IgL1-2. Then we measured the molecular conformation via Small-Angle X-ray Scattering (SAXS) in a similar manner as reported^28^ in order to verify the trans-homophilic dimerization of PECAM-1 in solution. Data analysis yielded a linear Guinier curve and a distance distribution indicating a rod-like shape (Sup Fig. 1A,B).

**Figure 3:**
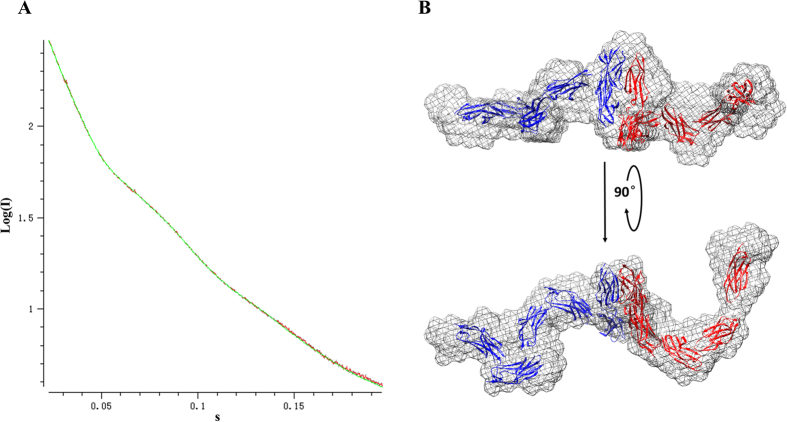
SAXS analysis of PECAM-1 IgL1-6 in solution. (**A**) The fitness between the scattering of calculated model (green curve) and experimental intensities (red curve with individual bar). (**B**) Low resolution envelope fitted with the crystal structures of IgL1-2 dimer and IgL1. Two different IgL1-6 molecules are in blue and red color respectively. Central part is assembled with an IgL1-2 trans-homodimer. Expanded arms with relative flexibility are fitted with eight IgL1 monomers.

Low resolution envelope was generated based on the bead model calculated from SAXS data. The scattering from the bead model was consistent with experimental intensities (Fig. 3A). The calculated envelope had adequate space to fit in two IgL1-6 molecules. The elongated envelope consisted of a large center that appeared to be an interaction region and two slender outstretched arms representing dissociative parts (Fig. 3B). Moreover, the envelope can be well fitted using the crystal structure of one PECAM-1 IgL1-2 dimer solved in this study for the central part and eight single IgL1 (to fit the IgL3-6 region where the crystal structure is unavailable) for the outstretched part. The SAXS model of PECAM-1 demonstrated that two PECAM-1 IgL1-6 molecules could form a trans-homophilic dimer in solution, which further validated the interaction of IgL1-2 determined in our crystal structure. Compared to IgL1-2, which can form a homo-dimer, IgL3-6 is much more flexible due to the absence of transmembrane and cytoplasmic portions which can anchor IgL3-6 on cell membrane.

### Cell adhesion is independent of glycosylation on IgL1-2

**Figure 4:**
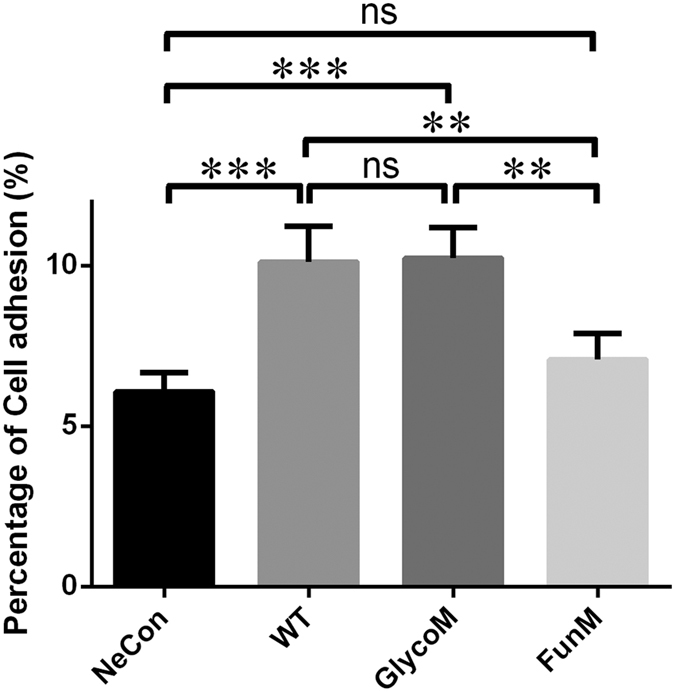
Mutational analysis on PECAM-1 mediated cell adhesion. Compared to negative control (NeCon) group, both wildtype (WT) and glyco-mutated (GlycoM, N52Q/N84Q/N151Q) PECAM-1 can significantly increase cell junction among HEK293 cells. Functional mutation (FunM, L74E/I112E/F188E/I190E) on PECAM-1 can decrease the probability of cell contact. (**P < 0.005; ***P < 0.0005; ns: non-significant).

PECAM-1 is a glycoprotein with nine potential glyco-sites on six IgL domains in which three of them localize on IgL1-2 with N52, N84 on IgL1 and N151 on IgL2. On account of differences between insect and mammalian cell protein expression systems, we used human derived HEK293 cell line to mimic the original expression environment of PECAM-1. For a full-length PECAM-1 glycosylation mutation sample, we mutated the same three sites in the IgL1-2 fragment as that chosen for structural study. Meanwhile, for a functional mutation sample, four residues (L74, I112, F188 and I190) situated in the hydrophobic center identified in our crystal structure were mutated to E to disrupt the intermolecular binding of PECAM-1. Then we inserted wildtype and mutated DNA sequences into mammalian transient expression vector and transfected into HEK293 cell line for cell adhesion assay. Transfected cell line would express full length PECAM-1 linked with C-terminal 3*FLAG tags derived from vector, and we used FLAG tag antibody to detect target protein expression. The principle of cell adhesion assay is based on PECAM-1’s ability to promote cell junction. Experimental subjects contain four groups: negative control (NeCon), wildtype (WT), glycosylation mutation (GlycoM) and functional mutation (FunM) groups constituted by cell lines transfected with empty, wildtype, glyco-mutated and functional mutated PECAM-1 vectors respectively. Each group was split into two equal portions. For labelling cell membrane, one portion was incubated with PKH26 red fluorescent cell linker, and the other incubated with PKH67 green fluorescent cell linker. We then mixed the two together in equal proportions and incubated them at 37 degrees for 30 mins. During the incubation, cell adhesion would happen. After that, we used flow cytometry to detect the percentage of cell adhesion between PKH26- and PKH67-cells. After using unlabeled cells and single labeled cells for gating in flow cytometry, PE and FITC areas represent PKH26- and PKH67-cells respectively, whereby Unstain area shows unlabeled cells and Adhesion area represents the cell junction between two different labeled cells. We counted the cell quantity in Adhesion area and calculated its percentage for quantification. Through the results (Sup Fig. 2), we found that cells with two different labels contacted with each other in a higher proportion in both WT and GlycoM groups than in the NeCon and FunM groups (n = 4). Either wildtype or glyco-mutated PECAM-1 can increase cell adhesion two-fold in WT and GlycoM groups, compared with NeCon and FunM groups (Fig. 4). Mutations on three potential glyco-sites did not affect physiological function of PECAM-1 on cell membrane. Furthermore, four residues (L74, I112, F188 and I190) important for hydrophobic binding can effectively influence PECAM-1 derived cell conjunction. Mutations on these four sites can effectually reduce trans-homophilic adhesion between PECAM-1-overexpressing HEK293 cells, which further certifies the homophilic binding interface of PECAM-1 IgL1-2 *in vivo*. Hence, with contribution of homophilic interaction of its IgL1-2, PECAM-1 derived cell adhesion is not glycosylation (N52, N84 and N151) related.

## Discussion

PECAM-1 participates in the formation of a strong barrier with other adhesion molecules between blood vessel and basal lamina. It also functions as a regulator and cooperator in the leukocyte trafficking process through both homophilic and heterophilic interaction on its extracellular region containing six Ig-like domains. The first two Ig-like domains (IgL1-2) can dimerize in a trans-homophilic manner which still has not been characterized to date. As a result, our crystal structure of PECAM-1 IgL1-2 trans-homophilic dimer not only shows a detailed drawing of Ig-like domains but also elaborates on the dimerizing mechanism of IgL1-2. As domains in a member of the Ig-superfamily, IgL1 and IgL2 of PECAM-1 have folds highly resembling those of traditional Ig domains. Each domain can be described as a “sandwich” architecture built with two β-sheet layers interlinked by a disulfide bond. The two tandem Ig-like domains cooperate with another homo-molecule via hydrophobic interaction and hydrogen bonds to form a tight trans-dimer. This trans-homophilic interaction was validated by small-angle X-ray scattering data of full-length wild type extracellular domain of PECAM-1. According to the cell adhesion assay, PECAM-1 derived cell junction is not associated with glycosylation on the first three putative N-glyco sites, which is consistent with our crystal structure of IgL1-2 possessing interface where two potential but uncertain glyco-sites (N84 and N151) localize.

A recent study reported a crystal structure of selenomethionyl IgL1-2 cis-homophilic dimer resulting from a “bridge” contact constituted by the last two β strands on each IgL2^26^. The last portion of IgL2 spanning residue 214–228, which interplays with the 12^th^, 13^th^ and 15^th^ β strands in the same molecule to form one β-sheet layer in our crystal structure, dissociates from its own IgL2, swings out and inserts into the corresponding position of IgL2 of a neighboring molecule, forming cis-homophilic IgL2. Although interfaces among IgL1-2 molecules were also identified through crystallographic symmetric operations, they did not contribute much to the binding. No interface was found in one asymmetric unit except the “bridge” contact. In contrast, our structure shows trans-homophilic dimerization of native IgL1-2 in one asymmetric unit, and the last peptide chain 214–228 in IgL2 does not swing out from its own architecture (Sup Fig. 3).

In this work, we have demonstrated the trans-homo-interaction mechanism of PECAM-1 IgL1-2. However, to fully understand the homophilic interaction of PECAM-1 and its function in cell junctions, the structure of full length or whole extracellular region of PECAM-1 needs to be determined. Our structure provides insights into understanding these multiple functions of PECAM-1 in endothelium integrity, immune response, and diapedesis.

### Data deposition

Coordinates and structure factors have been deposited in the protein data bank, under PDB code 5GNI.

## Material and Methods

### Vector cloning and mutagenesis

Plasmid that contained human PECAM-1 full length (FL, 1-738) DNA sequence was purchased from Addgene and IgL1-2 (28-232) DNA sequence suitable for insect cell expression with glyco-mutation (N52Q, N84Q and N151Q) was synthesized by GenScript. For recombinant protein expression, IgL1-6 (28–600) amplified by Polymerase Chain Reaction (PCR) from PECAM-1 FL DNA sequence and IgL1-2 digested from synthesized DNA sequence were cloned into pMib-V5-HisA vector (Invitrogen). IgL1 (28-132) and IgL2 (133–232) were amplified from synthesized DNA sequence and inserted into pET22b vector (Novagen). For cell adhesion assay, human PECAM-1 FL without stop codon was cloned into pCMV-3Tag-3A vector. Based on wildtype pCMV-3Tag-3A-PECAM-1 FL vector, GlycoM vector with mutations (N52Q, N84Q and N151Q) was produced by Site Directed Mutagenesis method point by point and FunM vector with mutations (L74E, I112E, F188E and I190E) was synthesized by BGI. All constructed vectors were validated by sequencing at BGI.

### Protein expression, purification and crystallization

For insect cell expression, pMib-V5-HisA vectors containing PECAM-1 fragments were transfected into High Five cell lines. Stable cell lines expressing target proteins were selected by blasticidin. Cells were cultured in High Five Express media (Invitrogen). Suspension culture volume was enlarged under 27 degrees. After 9–10 days, insect cell culture was collected and centrifuged at 4000 rpm under 4 degrees for 1 hour for removing cell debris. Supernatant was loaded onto Ni-column (GE), and target proteins were eluted by buffers with gradient concentration of imidazole step by step. For the purification of IgL1-6, eluted sample was concentrated directly and then loaded onto Superdex 75 column (GE). Target fractions were pooled and concentrated for SAXS. For IgL1-2, eluted sample was purified by ion-exchange (Q-HP, GE) and gel-filtration (Superdex 75, GE) columns. Target protein was concentrated for crystal screening based on commercial kits from Hampton Research. For *E. coli* cell expression, pET22b vectors with IgL1 and IgL2 were transfected into BL21 (DE3) Origami strain. Cells were cultured in 2*YT media and enlarged under 37 degrees. Protein expression was induced by 0.3 mM IPTG under 16 degrees for 18 hours. Cell culture was centrifuged at 4000 rpm under 4 degrees for 30 mins. After removing culture media, cell pellet was re-suspended in buffer (20 mM Tris-HCl pH = 8.0, 500 mM NaCl and 10 mM imidazole) and lysed by sonication. Lysed sample was spun down at at 17000 rpm under 4 degree for 30 mins. Similar to IgL1-2, IgL1 and IgL2 were purified and concentrated for Crystal screening. SeMet incorporation of IgL1 was performed as described previously. SeMet-IgL1 was purified in same way as native IgL1.

IgL1-2 (14 mg/ml), IgL1 (20 mg/ml) and IgL2 (18 mg/ml) were used for primary crystal screening through vapor diffusion method. We obtained IgL1-2 and IgL1 crystals but not IgL2. Crystal optimizations were based on best hits (0.1 M MES pH6.5/1.8 M (NH_4_)_2_SO_4_/0.01 M CoCl_2_ for IgL1-2 and 2.0 M (NH_4_)_2_SO_4_ for IgL1). IgL1-2 single crystals were produced in conditions (0.1 M MES pH6.2–6.8/1.4–1.6 M (NH_4_)_2_SO_4_/0.01 M CoCl_2_) through seeding method. IgL1 single crystals grew in condition (1.6–1.8 M (NH_4_)_2_SO_4_). The single crystals of Se-Met-IgL1 were harvested in the same conditions as native IgL1.

### Data collection, processing and structure refinement

Crystals were frozen in liquid nitrogen with cryo-protection. Diffraction data sets were collected at 100 K on BL17U beamline in Shanghai Synchrotron Radiation Facility (SSRF) and processed by *HKL2000*^29^. Phases of Se-Met-IgL1 data set were determined through single-wavelength anomalous dispersion (SAD) method with *AutoSol* in the Phenix program suite^30^. Model rebuilding was carried out by AutoBuild in Phenix program suite^30^. We then used the partial structure of Se-Met-IgL1 as a search model for molecular replacement on the IgL1-2 data sets with *Phaser* in the CCP4 program suite^31^. Subsequently, we manually rebuilt IgL1-2 model in *COOT* followed by refinement via the *Phenix_refine* program^30^. After many rebuilding-refinement cycles, we obtained the crystal structure of IgL1-2. Table 1 shows the crystallographic statistics of IgL1-2 and IgL1 data sets.

### Small-angle X-ray scattering

Purified PECAM-1 IgL1-6 and purification buffer that acted as target sample and solution background respectively were taken to SSRF for SAXS data collection on BL19U2 Bio-SAXS beamline. Collected image data was converted to.dat file by *BioXTAS RAW* software^32^. Further data processing, averaging and subtracting were accomplished by *Primus* in the ATSAS program package^33^. After determining the linear Guinier region and distance distribution to obtain extrapolated I_0_ and reasonable D_max_ respectively, bead modeling was carried out in slow mode with *Damif* called by *Primus*. The mesh envelope of final solution was represented by *Chimera* and fitted with the crystal structure of IgL1-2 and IgL1^34^.

### Cell adhesion assay

pCMV-3Tag-3A empty and PECAM-1 FL genes (WT, GlycoM and FunM) inserted vectors were transfected into HEK293 cell lines cultured in 10 cm dishes. Cells with overexpression of exogenous proteins were collected in two days. After removing culture media through centrifugation, cells was washed with PBS two times for eliminating residual FBS and then resuspended in 200 ul Dilute C buffer (SIGMA). Cell suspension was divided into two parts with same volume (100 ul). At the same time, 2 ul PKH67 green and 2 ul PKH26 red fluorescent cell linkers were diluted in 100 ul Dilute C buffer respectively. Then two homologous cell suspensions were mixed with cell linkers diluents and incubated in dark at room temperature for 5 mins followed by adding equal volume of FBS for terminating cell label processes. Centrifugation was performed under 4 degrees at 300 g for 5 mins. Labeled cells were collected and washed by PBS one time. Next, labeled cells were re-suspended by 300 ul PBS and counted. We mixed two equal amounts of labeled cells and incubated them in waterbath under 37 degrees for 30 mins. Adhered, unstained and single stained cells were analyzed by flow cytometry. FACS data sets were analyzed by FlowJo software.

## Additional Information

**How to cite this article**: Hu, M. *et al*. Structural Basis for Human PECAM-1-Mediated Trans-homophilic Cell Adhesion. *Sci. Rep.* **6**, 38655; doi: 10.1038/srep38655 (2016).

**Publisher's note:** Springer Nature remains neutral with regard to jurisdictional claims in published maps and institutional affiliations.

## Supplementary Material

Supplementary Information

## Figures and Tables

**Table 1:** Crystallographic Data and Refinement Statistics.

Data Collection		
	IgL1-2	IgL1
Cell dimensions		
a, b, c (Å)	60.5, 141.5, 169.9	97.5, 97.5, 128.3
α, β, γ (°)	90, 90, 90	90, 90, 90
Space group	I222	P2_1_2_1_2_1_
Wavelength (Å)	1.0719	0.97980
Resolution (Å)^a^	50.00-3.00 (3.05- 3.00)	50.00-2.00 (2.07- 2.00)
Unique reflections	14823 (709)	82445 (7952)
Redundancy	7.9 (8.0)	14.6 (13.0)
<I /σI>	25.12 (3.07)	28.91 (14.82)
Rsym	0.086 (0.838)	0.096 (0.187)
Completeness (%)	99.7 (100.0)	99.60 (97.03)
**Refinement**		
Rwork	0.22	
Rfree	0.28	
RMSD from ideal		
Bond lengths (Å)	0.01	
Bond angles (°)	1.35	
Average B-factors	64.4	
Ramachandran plot (%):	favored/allowed/outliers	94.0/6.20/0.26

^a^Values in parentheses are from the highest-resolution shell.
