# Supplementary material for: Structural Basis for Human PECAM-1-Mediated Trans-homophilic Cell Adhesion
Source: Sci Rep. 2016 Dec 13;6:38655. doi: 10.1038/srep38655 (PMC5153848; doi:10.1038/srep38655)

**Supplementary materials**

**Structural Basis for Human PECAM-1-Mediated Trans-homophilic Cell Adhesion**

Menglong Hu,^a^ Hongmin Zhang^b^, Qun Liu^c^, Quan Hao^a*#^

^a^ School of Biomedical Sciences, University of Hong Kong , Laboratory Block, 21 Sassoon Road, Pokfulam, Hong Kong, China;

^b^ Department of Biology and Shenzhen Key Laboratory of Cell Microenvironment, Southern University of Science and Technology, Shenzhen 518055, China.

^c^ Biology Department, Brookhaven National Laboratory, Upton, NY 11973, USA

^*^ To whom correspondence may be addressed: E-mail:qhao@hku.hk

^#^Contact number: +852-6228 0468

Keywords: PECAM-1, CD31, trans-homophilic dimer, cell adhesion, X-ray crystallography

**Sup. Figure 1. Validation of SAXS data.** (A) Guinier linear region identified by Primus at low q area for extrapolating I_0_. Plots involved in nonlinear and beyond s=0.2 were removed. (B) Distance distribution based on extrapolating I_0_ was generated by Primus and represents elongated shape of PECAM-1 IgL1-6 in solution.


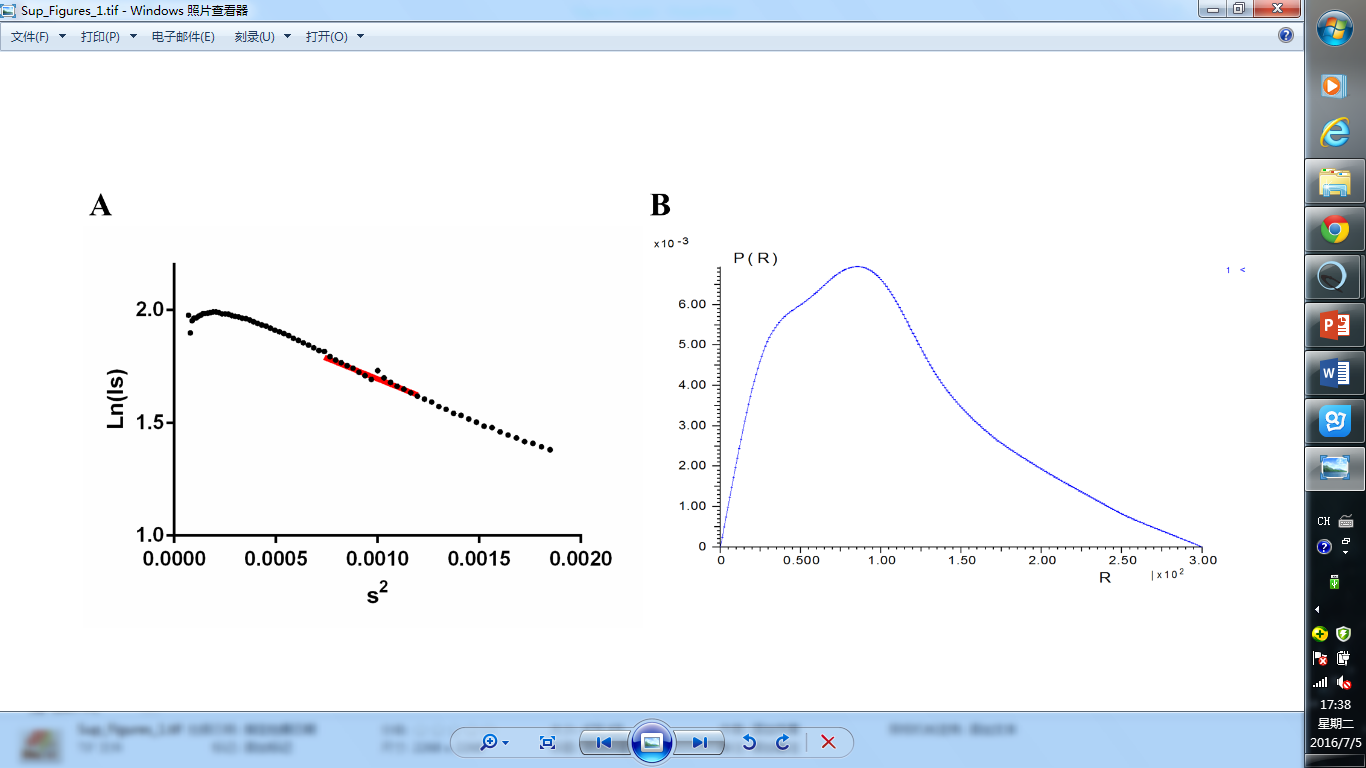


**Sup. Figure 2. Cell adhesion assays.** Unstain (without fluorescence label), PKH67 (with fluorescence label detected under PE filter setup) and PKH26 groups (with fluorescence label detected under FITC filter setup) are used for gating non-labeled and single labeled cells as controls. Groups encircled by black polygons are named Unstain, FITC and PE respectively. Cell percentages are calculated by FlowJo and recorded in each group. Adhesion area indicates where cell junction happens after mixture of cells with different fluorescent parts and its cell percentage should be counted as nearly zero in Unstain, PKH67 and PKH26 groups. Negative control (NeCon), wildtype (WT), glycosylation mutation (GlycoM) and functional mutation (FunM) groups are experimental participants. Each of them contains equal amount of cells with different fluorescence parts. After counting percentage at Adhesion area of each group, comparison among experimental groups and data statistics are calculated by *GraphPad Prism*.


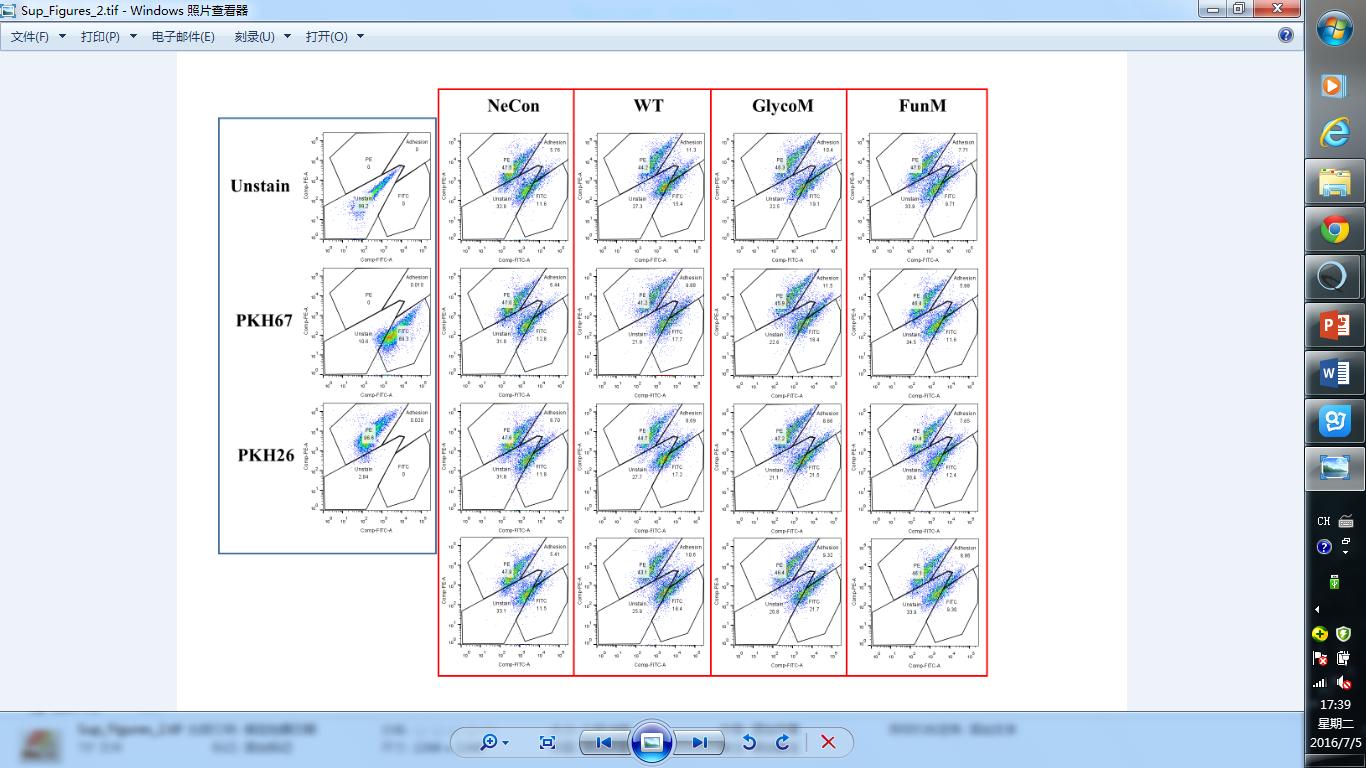


**Sup. Figure 3. Comparison between individual IgL1-2 in our structure and PDB 5C14**. Trans- (A) and cis- (B) homophilic dimers of IgL1-2 with colored IgL1 (blue) and IgL2 (red) domains. Green portions highlighted by red arrows are differences between intact (A) and swapped (B) IgL2 domains.


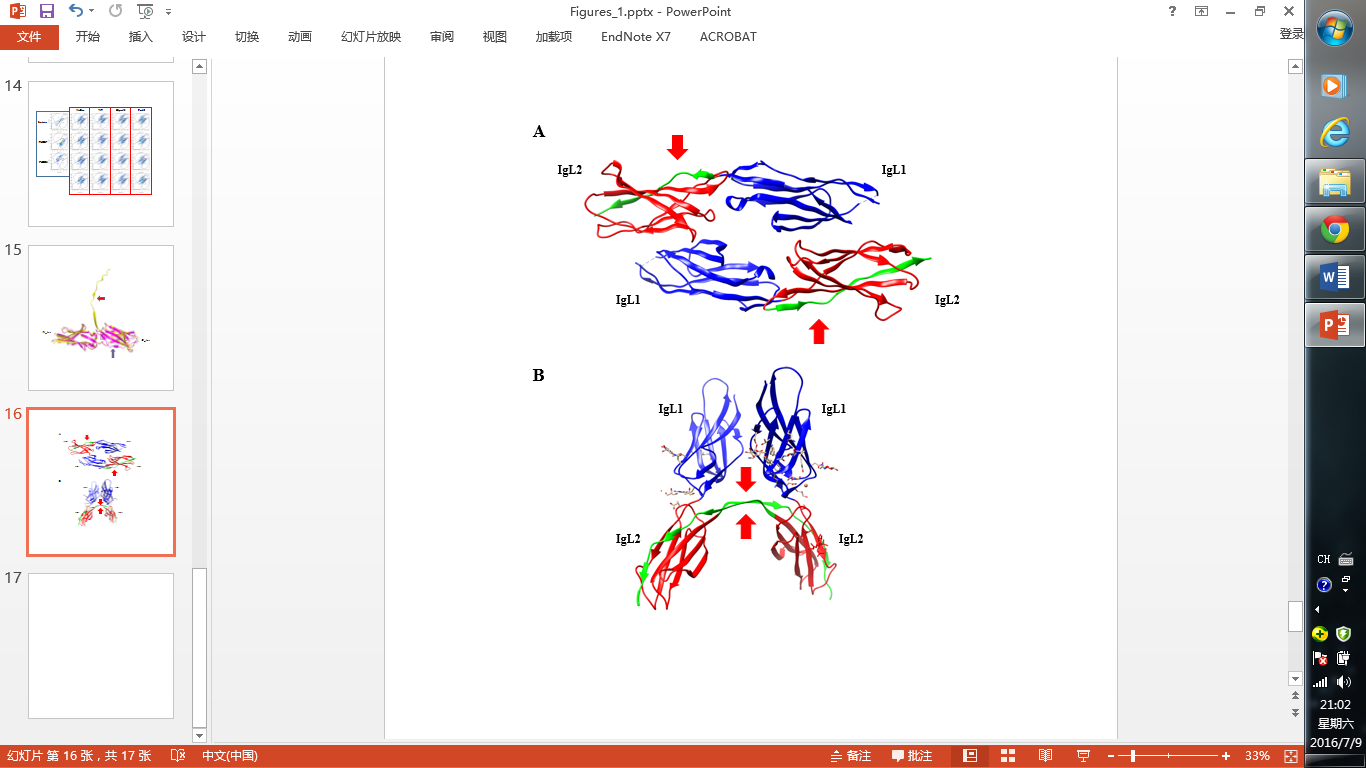

Supplement: Supplementary Information [file srep38655-s1.doc]
